# Cut-down assisted percutaneous gastrostomy tube placement in morbidly obese patient

**DOI:** 10.1093/jscr/rjac083

**Published:** 2022-03-30

**Authors:** Unnati Vishwakarma, Ahmad Hlayhel, Franz S Yanagawa

**Affiliations:** St. George’s University, School of Medicine, University Centre Grenada, West Indies, Grenada; Department of Surgery, St. Joseph’s University Medical Center, Paterson NJ, USA; Department of Surgery, St. Joseph’s University Medical Center, Paterson NJ, USA

## Abstract

Enteral and parenteral nutrition is primarily indicated in patients that lack adequate oral intake to support their metabolic needs. Percutaneous endoscopic gastrostomy (PEG) has become the preferred procedure of choice. With the increasing prevalence of obesity in the USA, there is a need for special interventions for PEG tube placements in overweight and obese patients. Some challenges that frequently arise with obese patients include sub-optimal transillumination, insufficient abdominal landmarks and inability to estimate the abdominal and gastric walls. We present a case of a patient with persistent dysphagia requiring enteral nutrition with an unconventional placement of a PEG tube given patient’s large body habitus.

## INTRODUCTION

Enteral and parenteral nutrition is primarily indicated in patients that lack adequate oral intake to support their metabolic needs. Enteral nutrition has been the preferred choice over parenteral nutrition in patients that have a functional gastrointestinal (GI) system because of the advantage of enteral stimulation that is otherwise lost with parenteral nutrition [1]. Selection of a device for enteral access depends on the patient’s disease status, anatomy, gastrointestinal mobility, function status and length of required therapy. A nasogastric tube is preferred when the patient requires nutrition for a short-term period whereas a gastrostomy/gastrojejunostomy is preferred for patients with long-term need for enteral nutrition (>4 weeks) such as cases of persistent dysphagia [2]. Common techniques for a gastrostomy tube placement include percutaneous endoscopic gastrostomy, laparoscopic-assisted and open surgical placement. Percutaneous endoscopic gastrostomy (PEG) has become the preferred procedure of choice due to lower costs, safety profile and decreased requirement for general anesthesia [3]. We present a case of a patient with persistent dysphagia requiring enteral nutrition with an unconventional placement of a PEG tube given patient’s large body habitus.

## CASE REPORT

The patient is a 49-year-old right-handed woman with a past medical history of morbid obesity, HIV, asthma and hypertension who presented to the emergency department after acute onset of left hemiparesis. Patient was found to have a hemorrhagic stroke of the right basal ganglia. After failing multiple swallow evaluations for dysphagia, the general surgery team was consulted for PEG placement.

Patient underwent attempted PEG placement in the operating room where transillumination during the procedure was successful, however, the procedure was aborted as the access needle was too short for the patient’s thick abdominal wall and the gastric cavity could not be accessed. The patient was later taken to the operating room for a second attempt with planning for laparoscopic assistance if needed. During the second attempt, transillumination was also achieved but due to large body habitus, the introducer needle was unable to traverse the abdominal wall to gain access to the gastric cavity. Subsequently, a 5-inch needle and a 7-inch spinal needle were also utilized with failure to gain access. At this point, a decision was made to attempt a cut-down assist at the transilluminated site. A 5-cm incision was made over the transilluminated site and the underlying subcutaneous fat was dissected and retracted until the fascia was visualized. Transillumination was noted on the fascial wall and a 5-inch needle and catheter were used to gain access to the gastric cavity. The needle was directly visualized through the endoscope. PEG tube was placed at this insertion site using standard pull technique without complication. The bumper was visualized with endoscopy abutting the stomach wall. The external portion of the PEG tube was noted to be at 2 cm at the skin. The skin incision was closed using 4–0 interrupted Monocryl suture on either end of the PEG tube and the tube was secured to the abdominal wall the external bumper and 2–0 Prolene suture.

## DISCUSSION

With the increasing prevalence of obesity in the USA, there is a need for special interventions for PEG tube placements in overweight and obese patients. Some challenges that frequently arise with obese patients include sub-optimal transillumination, insufficient abdominal landmarks and inability to estimate the abdominal and gastric walls [4]. Patients with a thick abdominal wall face challenges in PEG tube placement due to excess fat and lead to unsuccessful tube placement [5]. This demonstrates a need for an alternate method for successful PEG tube placement in obese patients for their large abdominal girth. The presented article introduces a novel method to place a gastrostomy tube in patients after appropriate transillumination is achieved. A similar approach was proven to be successful where a 5-cm transverse incision was used, fatty tissue was separated and the needle was passed through, to place a PEG tube in an obese patient [6].

## CONCLUSION

A laparoscopic or open approach is considered for gastrostomy tube placement by physicians when percutaneous approach fails. However, PEG tube placement is shown to have a lower risk of complications as compared to the open gastrostomy method as well as shorter post-operative stays and economical cost [7, 8]. In this case report, we provide an alternative method for PEG tube placement for overweight and obese patients that successfully achieved transillumination but failed PEG placement due to their large abdominal girth.

## CONFLICT OF INTEREST STATEMENT

None declared.

## FUNDING

None.

## DATA AVAILABILITY

The authors declare that data supporting the findings of this study are available within the article.

## AUTHORS’ CONTRIBUTIONS

UV and AH led the drafting of the manuscript. FY and AH edited the manuscript and have responsibility for its final content.
